# Ultrasound-Assisted Extraction of *Nannochloropsis oculata* with Ethanol and Betaine: 1,2-Propanediol Eutectic Solvent for Antioxidant Pigment-Rich Extracts Retaining Nutritious the Residual Biomass

**DOI:** 10.3390/antiox11061103

**Published:** 2022-05-31

**Authors:** Maria D. Gkioni, Vasilis Andriopoulos, Eleni Koutra, Sophia Hatziantoniou, Michael Kornaros, Fotini N. Lamari

**Affiliations:** 1 Department of Pharmacy, School of Health Sciences, University of Patras, 26504 Patras, Greece; marigion00@gmail.com (M.D.G.); sohatzi@upatras.gr (S.H.); 2 Department of Chemical Engineering, School of Engineering, University of Patras, 26504 Patras, Greece; billandri@upatras.gr (V.A.); ekoutra@chemeng.upatras.gr (E.K.); kornaros@chemeng.upatras.gr (M.K.)

**Keywords:** microalgae, *Nannochloropsis oculata*, analysis, carotenoids, fatty acids, eicosapentaenoic acid, antioxidant properties, extraction

## Abstract

The aim of this study was the development of an efficient “green” extraction method of *Nannochloropsis oculata* to produce antioxidant extracts and nutritious residual biomass. Twenty-one extraction methods were evaluated by measuring the reactivity with the Folin–Ciocalteu reagent: ultrasonication or maceration at different temperatures with different organic solvents, extraction at different pH values, enzyme-assisted extraction, encapsulation with β-cyclodextrin, and the use of natural deep eutectic solvents. Ultrasound-assisted extraction with ethanol or betaine: 1,2-propanediol in a molar ratio of 2:5 (BP) had optimal extractive capacity. Both extracts were evaluated with antioxidant assays and the ethanol extract exhibited significantly higher (at least twofold) values. The determination of carotenoids by LC-MS and HPLC-DAD revealed the dominance of violaxanthin and antheraxanthin and their fourfold higher concentrations in the ethanol extract. The ^1^H-NMR characterization of the ethanol extract confirmed the results of the colorimetric and chromatographic assays. The microalgal biomass was characterized before and after the extraction in terms of humidity, ash, carbohydrates, proteins, chlorophyll-a, carotenoids, and lipids; the identity and content of the latter were determined with gas chromatography. BP caused a smaller depletion of the lipids from the biomass compared to ethanol, but proteins, carbohydrates, and ash were at a higher content in the biomass obtained after ethanol extraction, whereas the biomass was dry and easy to handle. Although further optimization may take place for the scale-up of those procedures, our study paves the way for a green strategy for the valorization of microalgae in cosmetics without generating waste, since the remaining biomass can be used for aquafeed.

## 1. Introduction

Microalgae are an extremely diverse group of microorganisms that include prokaryotic cyanobacteria and eukaryotic photosynthetic organisms. Their utilization by humans has a long history; native populations in Mexico, Chad, China, and Mongolia have traditionally consumed *Arthrospira* and *Nostoc* species due to their high content in proteins and lipids [1]. Meanwhile, thousands of tons of *Chlorella* and *Arthospira* (2000 and 5000, respectively) are produced every year in order to be drained as food supplements at the market [2]. Lipids (mainly with polyunsaturated fatty acids), proteins (with essential amino acids), polysaccharides (β-glycans), vitamins, pigments (chlorophylls, carotenoids, and phycobiliproteins), polyphenols, and phytosterols are some of the valuable metabolites in microalgae [1,2,3]. Those ingredients have great nutritional value and confer many health benefits not only to humans. Recently, microalgae have been used as animal and fish feed [1,2] due to their valuable ingredients and the environmental sustainability of their production. Marine microalgae cultivation has several advantages over terrestrial crops, such as improved land and water use efficiency because of higher yields per unit input without the need for freshwater or arable land, and reduction in carbon emissions [4]. 

The genus *Nannochloropsis* (Monodopsidaceae family, Eustigmatophyceae class) was described by Hibberd (1981) and contains microalgae that are found in fresh, brackish, and ocean waters. Those are one-cell, almost spherical organisms with a diameter <5 μm. Those green eukaryotic microalgae are well-known for their high growth rate and their oleaginous biomass and are suitable for industrial-scale cultivation. *Nannochloropsis* produces many nutritionally valuable mono- and poly-unsaturated fatty acids; among them, eicosapentaenoic acid (EPA) is in remarkable amounts (>2% dry weight) [1]. *Nannochloropsis* sp. lacks chlorophyll-b and -c and has high amounts of chlorophyll-a, which is the main pigment [5,6]. In the Eustigmatophycean algae, violaxanthin is the epoxy-carotenoid with a major role in photosynthetic light harvesting [7]. Other *Nannochloropsis* carotenoids are antheraxanthin, vaucheraxanthin, zeaxanthin, neoxanthin, and β-carotene, as well as smaller quantities of the keto-carotenoids echinone, castaxanthin, astaxanthin, astaxanthin’s monoester, and astacene [5,6,8,9,10].

Biomass or extracts of *Nannochloropsis* sp. have many industrial applications. The potential for manipulation of its biosynthetic machinery by genetic engineering and for the direction of the metabolism by an intricate choice of the cultivation conditions makes it very attractive for sustainable production [11]. The cosmetic applications of *Nannochloropsis* are under investigation [3,12]. In particular, a pigment extract of *Nannochloropsis oceanica* in human dermal fibroblasts resulted in significant inhibition of UVB-induced reactive oxygen species production, loss of cell viability, extracellular matrix degradation, and cellular senescence; violaxanthin was demonstrated as an antiphotoaging agent [13]. The propylene glycolic extract of *Nannochloropsis gaditana* demonstrated impressive antioxidant, anti-aging, healing, DNA protecting, and skin texture and hydration improving properties throughout a series of in vitro experiments [14]. In a later study, Kim et al. [15] demonstrated in vitro that the NG15 *Nannochloropsis* extract showed skin protective functions, i.e., low cytotoxicity, anti-melanogenic, antioxidant, skin-moisturizing, anti-inflammatory, anti-wrinkling, and UV protective function. Concerning its use in aquaculture, it not only provides important nutrients (lipids, proteins, and amino acids), but also displays bactericidal and bacteriostatic activity [16]. In order to minimize the cost of the final product for aquafeed and to valorize the co-/by-products created by the treatment of microalgae for other purposes, there is ongoing research on the effectiveness of using microalgal lipid-depleted co-products from *Nannochloropsis oculata* as fish meal and the results are very encouraging [17].

*Nannochloropsis* strains have fairly thick and robust cell walls consisting of a cellulosic inner wall protected by an outer hydrophobic layer [18]. Various mechanical, chemical and biological methods for cell disruption of microalgae have been suggested, including cryogenic crushing and grinding, electrical disruption ultrasonication, high-speed or high-pressure homogenization, freeze-thaw cycles, and enzymatic and thermal hydrolysis [18,19,20,21]. Cell disruption by the usage of ultrasonic power is an efficient and cost-effective method for the isolation of pigments [19,22]. Gallego et al. [8] reported that ultrasound-assisted extraction (UAE) along with freeze-thaw cycles were superior. The use of enzymes to degrade the cell walls has been investigated in a few studies with a focus on lipid extraction; Zuorro et al. [23] experimented with a combination of cellulase and mannanase at pH 4.4; Wu et al. [24] suggested alkaline pretreatment at pH 10.5 at 110 °C and then treatment with a mixture of cellulase, protease, lysozyme, and pectinase at pH 4.0; and Chen et al. [25] suggested thermal lysis followed by treatment with cellulase and protease, and then extraction with surfactants. None have investigated the effect of enzymic treatment on pigment extraction, whereas Safi et al. [26] used Alcalase^®^ for protein extraction (35%). Extraction of lipids and pigments may be performed with toxic organic solvents in a laboratory (e.g., chloroform, dichloromethane, and/or methanol), but the prerequisite of sustainable large-scale production is the use of green nontoxic solvents [27]. Supercritical fluid extraction has been employed especially for lipid extraction [28], whereas, with the addition of ethanol, most pigments are also extracted [29]. Ionic liquids and surfactant additives have been studied for their lipid extractive capacity [19], whereas Lee et al. [30] used 2,3-butanediol for the extraction of chlorophyll-a. Mehariya et al. [31] stressed the potential of natural deep eutectic mixtures (NaDES) for the extraction of bioactives from microalgae, especially in combination with sonication, but none have been applied to *Nannochloropsis* to our knowledge. Another strategy employed for the extraction of polyphenols from plant sources is the one-pot extraction and encapsulation with cyclodextrins [32,33], but this has not been tested in *Nannocloropsis*. Within the context of biorefinery, high-pressure homogenization and supercritical fluid extraction were applied as a first step to extract nonpolar lipids and pigments, and then pressurized liquid extraction with ethanol was used for the further valorization of the remaining residue [34].

Aligned with the ongoing efforts for the sustainable valorization of microalgae as a blue multi-product biorefinery [4,20], the primary goal of this investigation was to develop extracts of *Nannochloropsis oculata* with high antioxidant capacity for cosmetic use on the condition that the extracted biomass remains appropriate for aquafeed. For that reason, we embarked on the evaluation of different extraction methods using classic green solvents, such as ethanol, aqueous solutions at different pH values, neoteric solvents, such as NaDES, combinations of enzymes, and encapsulation with cyclodextrins; NaDES and cyclodextrins have not been tested earlier in *Nannochloropsis*. In this study, we evaluated a total of 21 different extraction methods of *N. oculata* with the Folin–Ciocalteu assay. The Folin-Ciocalteu reaction is based on electron transfer, and the assay measures the reductive capacity of a compound. At first, it was used for the determination of tyrosine but, nowadays, it is widely applied in the determination of the total phenol/polyphenol content or the antioxidant activity [35,36]. For the two superior methods, an in-depth evaluation of the biomass and the extracts was conducted. Humidity, ash, carbohydrates, proteins, chlorophyll-a, carotenoids, and lipids were determined in the biomass before and after the extractions. A chromatographic method was developed for the determination of the carotenoids in the extracts, whereas their antioxidant capacity was assessed with ferric reducing and radical scavenging assays.

## 2. Materials and Methods

### 2.1. Nannochloropsis oculata Cultivation

*N. oculata,* provided by the Laboratory of Zoology (Department of Biology, University of Patras, Greece), was cultivated for 13 days in 6 glass vessels with an operational volume of 1500 mL using 4 times concentrated f/2 medium without silicate or vitamins. Temperature and pH were monitored and controlled with a Hach SC200 controller. The temperature was maintained at 21.0 ± 1.2 °C with an internal cooling coil and pH at 8.0 ± 0.2 with the addition of 1N HCl via a peristaltic pump. Continuous illumination of ~55.6 Lm was provided by one 1.5 m LED tape (CubaLUX, 6000K) wrapped around each vessel, between the 400 mL and 1500 mL marks. Aeration with ambient air at a rate of ~2.8 L L^−1^ min^−1^ was provided via a diffuser, while additional mixing was provided via magnetic stirrers at a rate of ~500 rpm. Initial biomass and chlorophyll-a concentrations were ~0.05 g L^−1^ dry weight (DW) and ~1.6 mg L^−1^, respectively. Optical density was measured with a Cary^®^50 UV/VIS spectrophotometer from Varian, Inc. (Palo Alto, CA, USA). Biomass density was measured as total suspended solids (TSS) according to *Standard Methods for the Examination of Water and Wastewater* [37] using 0.5 M ammonium bicarbonate as the washing solution. Nitrate and total phosphate concentration were monitored according to *Standard Methods for the Examination of Water and Wastewater* [37]. Biomass was collected via centrifugation at 15,000 rpm (Z 32 HK, Hermle AG, Gosheim, Germany) for 5 min at the onset of nitrate depletion when biomass and chlorophyll-a concentrations were 0.80 ± 0.14 g L^−1^ DW and 24.2 ± 2.6 mg L^−1^, respectively. Collected biomass was washed with 0.5 M ammonium bicarbonate, freeze-dried, pulverized, and stored in a desiccator until use.

### 2.2. Enzymes and Chemicals

Nutrients used for the cultivation of *N. oculata* were CoCl_2_.6H_2_O (Thermo Fisher Scientific, Pittsburg, PA, USA), CuSO_4_.5H_2_O (Sigma-Aldrich, St. Louis, MO, USA), FeCl_3_.6H_2_O (Acros Organics, Geel, Belgium), MnCl_2_.4H_2_O (Acros Organics), Na_2_EDTA (Sigma-Aldrich), Na_2_MoO_4_.2H_2_O (Chem-Lab NV, Zedelgem, Belgium), NaH_2_PO_4_.2H_2_O (Honeywell International Inc., Charlotte, NC, USA), NaNO_3_ (PanReac, Barcelona, Spain), and ZnSO_4_.7H_2_O (Sigma-Aldrich). Betaine, 1,2-propanediol, glycerol, glucose, urea, and β-cyclodextrin were purchased from Sigma-Aldrich. Chemicals used for analysis and evaluation of antioxidant activity were 1,1-diphenyl-2-picrylhydrazyl radical (DPPH) (Sigma-Aldrich), 2,2′-azinobis-(3-ethylbenzothiazoline-6-sulfonate (ABTS) (Sigma-Aldrich), 2,4,6-tri(2-pyridyl)-s-triazine (TPTZ) (Alfa Aesar, Ward Hill, MA, USA), acetic acid (PENTA, Prague, Czech Republic), ammonium bicarbonate (Sigma-Aldrich), chloroform HPLC grade (Honeywell), EDTA-Na_2_, 6-hydroxy-2,5,7,8-tetramethylchroman-2-carboxylic acid (Trolox) (Acros Organics), potassium persulfate (Acros Organics), Folin-Ciocalteu reagent (Sigma-Aldrich), hexane HPLC grade (Sigma-Aldrich), hydrochloric acid (HCl) (PENTA), iron (II) chloride anhydrous (Acros Organics), mercury (III) oxide red (Sigma), methanol/acetonitrile/acetone HPLC grade (Fischer Scientific), N,N-dimethylformamide (DMF) (Honeywell), sodium acetate trihydrate (Sigma-Aldrich), and water for analysis (Carlo Erba). Alcalase^®^ 2.4 L FG, a protease from *Bacillus licheniformis*, and Viscozyme^®^ L, a multi-enzyme cell-wall-degrading complex from *Aspergillus* sp. containing mainly endo-β-glucanase, along with a wide range of carbohydrases were purchased from Sigma-Aldrich.

### 2.3. Extraction

The dry biomass was subjected to various extraction procedures that are listed in Table 1. Enzymatic treatment and extraction with β-cyclodextrin and with NaDES were based on previous studies with minor modifications [32,33,38,39,40,41,42]. The NaDES, i.e., betaine: 1,2-propanediol in a molar ratio of 2:5 (BP), betaine: glycerol in a molar ratio of 1:2 (BG), betaine: glycerol: glucose in a molar ratio of 4:20:1 (BGG4), and urea: glycerol in a molar ratio of 1:1 (UG), were prepared by heating the mixtures to 80 °C with constant stirring until a homogeneous liquid formed.

### 2.4. Chlorophyll Determination

Chlorophyll-a was determined spectrometrically using a UV-spectrophotometer (UV-2401PC, Shimadzu Corporation, Kyoto, Japan) according to the equations of Lichtenthaler and Buschmann [43] for ethanol. The dilution of the extracts in ethanol was quite big, 1:500, so the equation was kept the same for the BP extract.

### 2.5. Ferric-Reducing Antioxidant Power Assay (FRAP)

The FRAP antioxidant assay measures the ability of antioxidants to reduce the [Fe(TPTZ)_2_]^3+^ to [Fe(TPTZ)_2_]^2+^ [44]. In particular, in a 96-well microplate, 10 μL sample or standard solution, followed by 190 μL FRAP reagent were placed. The FRAP reagent was freshly prepared by mixing 300 mM acetate buffer of pH 3.6, 10 mM TPTZ in 40 mM HCl, and 20 mM ferric chloride hexahydrate in a ratio of 10:1:1. The plate was incubated at 37 °C for 5 min. The absorbance was measured at 595 nm using a Sunrise^®^ microplate reader from Tecan Trading AG (Männedorf, Switzerland). Trolox was used as a standard. The results were expressed as mg Trolox equivalent per g of dry biomass (mg TEQ/g DB) based on the plotted calibration curve of the standard Trolox.

### 2.6. Radical Scavenging Activity Assay

For the evaluation of the radical scavenging activity of the samples, the scavenging of the DPPH and ABTS radicals were determined [45,46]. For the DPPH assay, 20 μL sample or standard solution (Trolox), was placed in a 96-well microplate. A total of 80 μL of 4 mM DPPH methanolic solution was added and, subsequently, the plate was kept for 30 min in a dark place at ambient temperature. The absorbance was measured at 540 nm. 

Regarding the ABTS assay, a stock ABTS solution was prepared by mixing 88 μL of 140 mM K_2_S_2_O_8_ with 7 mM ABTS solution in order to reach a volume of 5 mL. The stock solution was kept in a dark place for 12–16 h. After that, the ABTS stock solution was diluted with ethanol until it gave an absorbance between 0.680 and 0.720 at 734 nm. A total of 20 μL sample or standard solution (Trolox) was combined with 280 μL of ABTS solution in a 96-well microplate. The absorbance at 750 nm was measured immediately after a 5 min incubation at 30 °C. 

In both cases, the percentage of the radical scavenging activity was calculated by the equation %RSA=[Ablank−(Asample−Acontrol)/Ablank]×100. The results were expressed as mg Trolox equivalent per g of dry biomass (mg TEQ/g DB) based on the linear area of the plotted calibration curve of Trolox.

### 2.7. Folin-Ciocalteu Antioxidant Assay

The assay was based on Singleton & Rossi [47] with modifications. In a 96-well microplate, 20 μL sample or standard solution (gallic acid) was added, followed by 180 μL water and then 20 μL Folin reagent (1:10 dilution). The assay was completed with the addition of 20 μL 13.75% Na_2_CO_3_. The plate was kept in a dark place at ambient temperature for 30 min, and then absorbance was measured at 750 nm. Results were expressed as mg gallic equivalents per g of dry biomass (mg GAE/g DB). 

### 2.8. Chromatographic Determination of Carotenoids

LC-DAD-MS analysis was performed on a Dionex UltiMate 3000 UHPLC system (Thermo Fisher Scientific, Waltham, MA, USA) coupled to a quadrupole ion-trap Bruker amaZon SL MS equipped with an ESI interface (Bruker Daltonics GmbH & Co. KG, Bremen, Germany). The separation was performed on an Acclaim 120 C18 (2.1 mm × 100 mm, 3 μm) (Thermo Fisher Scientific). The flow rate was 0.3 mL/min, while the injection volume was 7 μL. The mobile phase consisted of 0.2% (*v*/*v*) formic acid in water (A) and 0.2% (*v*/*v*) formic acid in acetonitrile (B). The gradient elution started with 40% B, and, in 10 min, was up to 100% B and kept there until 30 min. The column was thermostated to 40 °C. For data processing, Bruker Compass Data Analysis V4.2 software (Bruker Daltonics GmbH & Co. KG) was used.

The quantification of the carotenoids of the *N. oculata* extracts was carried out on a Poroshell C18 column (250 mm × 4.6 mm, 5 μm). The chromatographic instrumentation consisted of a 1260 Infinity II HPLC (Agilent Technologies Inc., Santa Clara, CA, USA) coupled with a DAD detector and a manual injection valve from Agilent Technologies Inc. The column was thermostated at 30 °C and the flow was set at 0.7 mL/min. Four solvents were used for the elution: A: H_2_O containing 0.1% formic acid, B: methanol, C: acetonitrile, and D: methanol: acetone, 80: 20, *v*/*v*. The ratio A/B/C/D of the solvents at the different elution time points was as follows: 0 min: 55/5/40/0; 3 min: 5/5/90/0; 11 min: 5/5/90/0; 17 min: 0/10/90/0; 21 min: 0/10/90/0; 22 min: 0/0/0/100; and 45 min: 0/0/0/100. Data were processed with OpenLab Chemstation (Agilent Technologies Inc.).

Quantification was performed with astaxanthin as an external standard (≥97%, Sigma-Aldrich) at 430 nm with seven concentrations between 0.39 and 25.00 μg/mL. The calibration curve was y=84.686x−18.963, $R^{2}=0.9993$. The LLOD and LLOQ were 0.42 and 1.27 μg/mL, respectively.

### 2.9. NMR Characterization of the Ethanolic Extract

Τhe dry residue of the ethanolic extract was dissolved in CD_3_OD and transferred into a standard 5 mm NMR tube. NMR spectra were recorded using a Bruker AVANCE^®^ spectrometer operating at the ^1^H frequency of 600.13 MHz. ^1^H spectra of the extract were obtained using the following parameters: 300 K, 64 transients, 65536 data points, a spectral width of 12,019.23 Hz, recycle delay of 0.1 s, and a 30° flip angle pulse. Bruker TopSpin^®^ and MestreNova^®^ software were used for the NMR data analysis.

### 2.10. Biomass Characterization

Protein content was measured using the semi-micro Kjeldahl method [37], with a total Kjeldahl nitrogen-to-protein conversion factor of 6.25. The carbohydrate content was determined using the phenol-sulfuric acid method [48]. The lipid content and profile were determined using one-step in situ transesterification [49], and the resulting fatty acid methyl esters were analyzed using a GC (Agilent Technologies Inc., 7890A) equipped with a flame ionization detector and a capillary column (DB-WAX, 10 m × 0.1 mm × 0.1 m) [50]. The moisture content of freeze-dried biomass was tested after drying at 105 °C [37], and the ash concentration was determined after 45 min of incineration at 550 °C [37]. Total carotenoids and chlorophyll-a and -b were quantified by extracting with N,N′-dimethylformamide at 25 °C for 20 min, and then spectroscopically estimated based on previous research [51,52].

### 2.11. Statistical Analysis

Significant differences among the results of different treatments were evaluated with one-way analysis of variance and post-test Bonferroni multiple comparisons test with Graph Pad Instat 3 for Mac. A significance level of 5% was assumed for each analysis. The error bars presented in the figures correspond to the standard deviations.

## 3. Results and Discussion

### 3.1. Extraction Method Selection

**Figure 1:**
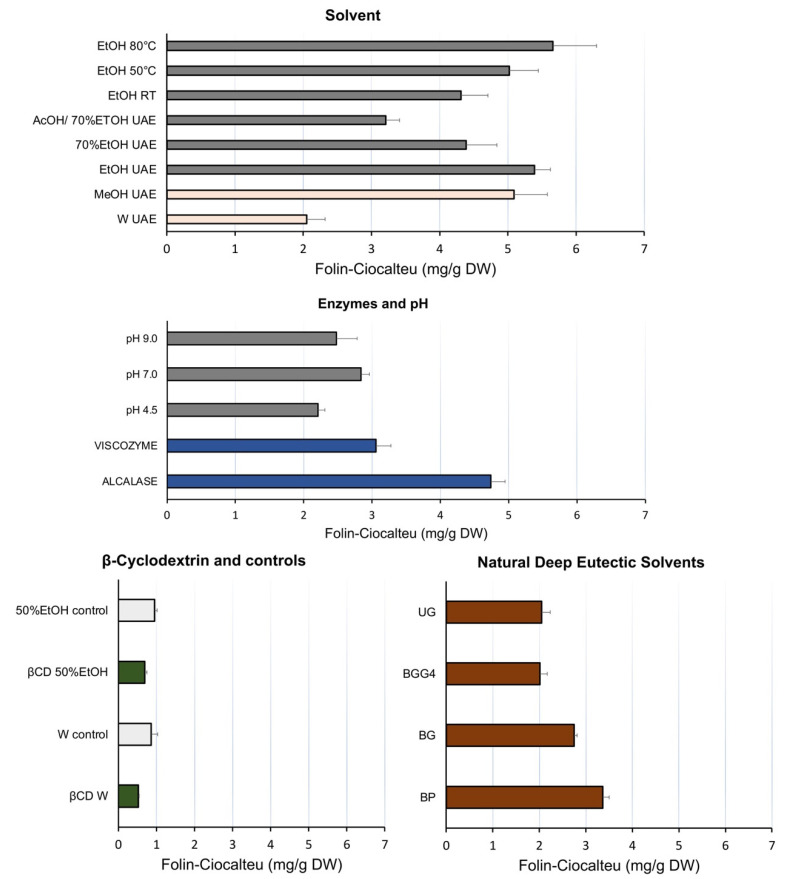
Comparison of the different extraction methods in terms of Folin–Ciocalteu reactivity. Results are expressed as mg gallic acid equivalents per g dry original biomass (*n* = 3).

The first factor that was assessed was the type of solvent. Pure methanol (MeOH UAE) and ethanol (EtOH UAE) were the best solvents in comparison to water, pure or acidified hydroethanolic solutions with comparable high values of GAE (5.09 ± 0.49 mg/g DW and 5.39 ± 0.23 mg/g DW, respectively) (Figure 1). Methanol, though not a green solvent, was assessed because it is commonly used in laboratory processes. Since the extracts are intended for cosmetic use and the residual biomass for aquafeed, ethanol was chosen for further examination as a green and less toxic solvent. Moving on to the evaluation of the role of temperature, maceration at 50 °C and 80 °C (EtOH 50 °C and EtOH 80 °C) gave comparable results with no statistically significant differences from ultrasound extraction at <40 °C (EtOH UAE). Therefore, UAE was selected as a less energy-consuming process and was adopted for the rest of the experiments. 

Experiments were conducted to evaluate if the organic solvent could be avoided, and thus the extraction at different pH values (4.5, 7.0, and 9.0) and in the presence of lytic enzymes was performed (Table 1, Figure 1). Alcalase^®^ (serine endopeptidase) catalyzes the hydrolysis of proteins at pH 7.0, whereas Viscozyme^®^ is a complex of carbohydrate-degrading enzymes, including arabinase, cellulase, β-glucanase, hemicellulase, and xylanase, operating at pH 4.5. Treatment with enzymes resulted in higher yields than their respective controls (pH 7.0 and pH 4.5). Alcalase^®^ was the most effective in GAE yield of the extract, not significantly different from ethanol (EtOH UAE), but the reactivity with Folin-Ciocalteu stems from the proteins degraded (Figure S1). When we extracted the remaining biomass with ethanol, the highest values of GAE were recorded (7.13 ± 0.41 mg/g GAE, not shown in Figure 1) but, since we wanted the biomass to remain nutritious for aquafeed, we abandoned that set of experiments. Encapsulation experiments (βCD 50% EtOH and βCD W) gave the lowest GAE values of all experimental sets, lower (*p* > 0.05) than their respective controls without β-cyclodextrin (50% EtOH control and W control); therefore, the study of β-cyclodextrin for one-pot extraction/encapsulation was abandoned. Other cyclodextrins of different size and hydrophilicity might be more appropriate.

Last but not least, among all NaDES, the BP solvent seemed to have potential, since its extract had 3.36 ± 0.14 mg GAE/g DW (Figure 1), not statistically significantly different from VISCOZYME and pH 7.0. BP is characterized by favorable properties, such as no toxicity, low cost, ingredients that can be used in foods and cosmetics, and being a green solvent. It was introduced by Mulia et al. [39] as an environmentally friendly solvent for extraction at mild conditions of a xanthonoid from the rind of mangosteen fruit. BP has polarity similar to that of ethanol, but much higher viscosity, which finally affected the extraction yield; another advantage of its use is the potential for heating during extraction [39]. Both BP and EtOH UAE were chosen for further analysis in order to investigate the potential of NaDES in *Nannochloropsis* extraction, although EtOH UAE was more efficient (*p* < 0.05).

### 3.2. Characterization of the EtOH-UAE and BP Extracts

Apart from the Folin–Ciocalteu assay, the antioxidant activity of the EtOH UAE and BP extracts was evaluated with the FRAP and the DPPH and ABTS radical scavenging assays. The results are presented in Table 2. The EtOH UAE extract was superior (*p* < 0.05) in all assays.

**Figure 2:**
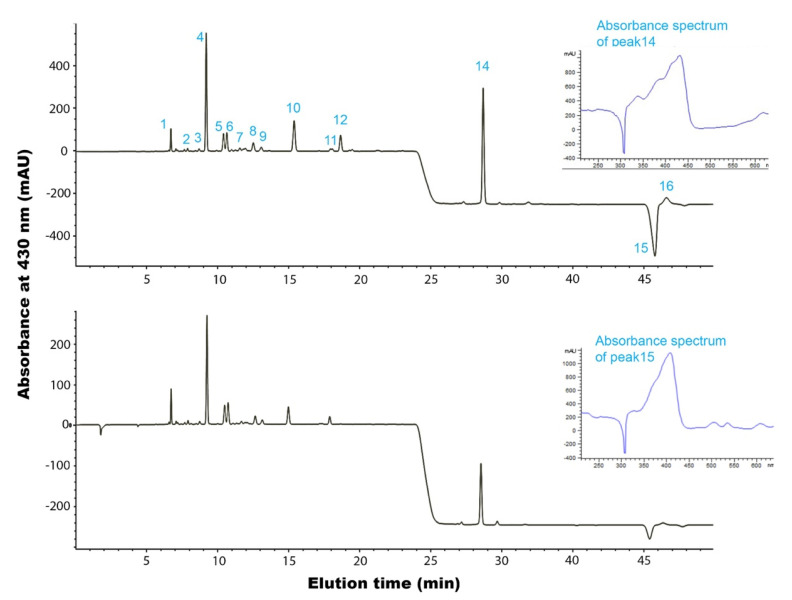
Typical HPLC chromatographs at 430 nm of the EtOH-UAE extract (upper) and the BP extract (lower). At this wavelength, phaeophytin (peak 15) appears as a negative peak. The UV-vis spectra of peaks 14 and 15 are shown. The peaks are numbered as in Table 3.

HPLC-DAD and UHPLC-DAD-MS methods with a good separation capacity of the main peaks were developed for pigment identification, whereas the quantification of carotenoids was carried out with HPLC-DAD (Figure 2). The original attempts of HPLC separation suffered from the lack of elution of chlorophyll-a and β-carotene from the C18 column and/or their significant carry-over from run to run. Acetonitrile alone was not a good eluent and, after pilot experiments, methanol and acetone were also added in a rather unusual interplay, but very efficient in terms of analytical repeatability (<2% and <15% relative standard deviation for retention times and peak areas after four repetitions of the sample on the same day) and lack of carry-over, as evidenced by blank injections after each analysis. Elution was performed isocratically from 3 to 11 min with 90/5/5 (acetonitrile/methanol/0.1% formic acid), and then a gradient of methanol from 5 to 10% with constant 90% acetonitrile was used from 11 to 17 min. Finally, elution was performed with methanol: acetone, 80: 20, *v*/*v* from 22 min to the end, causing a shift of the baseline. Separation was monitored at 430 nm due to the presence of pigments, whereas monitoring at 280 nm showed the absence of other compounds (Figure S2). With regards to quantification, the absence of commercial standards was a limitation that was overcome with the usage of astaxanthin (elution time: 11.4 min), a xanthophyll analogous to those previously reported for *Nannochloropsis*, and the results are expressed as astaxanthin equivalents.

In total, the extracts contained 16 pigments, i.e., 13 xanthophylls, 1 carotene, 1 chlorophyll-a, and 1 pheophytin-a, as shown in Table 3 and Figure 2. The identification of the compounds was carried out by considering their UV-vis maxima, elution order, and mass spectra in the positive ionization mode of MS. In certain cases (peaks 1, 2, 3, 5, 6, 7, and 12), the assignment as a derivative of a certain xanthophyll was heavily dependent on the absorption characteristics, as earlier suggested [8]. The carotenoids appeared as molecular or protonated molecular ions. The commonest fragments were [M−18+H]^+^ or [M−17]^+^ or [M+H−18−18]^+^ that are typical of carotenoids containing one or two hydroxyl groups (respectively), due to the loss of water molecules [53]. In luteoxanthin and auroxanthin derivatives, extra ions of [M−15]^+^ were recorded, which might reveal the loss of methyl groups.

The total amount of carotenoids was 24.50 and 5.53 mg astaxanthin equivalent/g DW in the ethanolic and BP extracts, respectively. Violaxanthin (peak 4) was indeed the most abundant ingredient, with 10.41 and 2.34 mg/g DW in ethanolic and BP extract, respectively. Antheraxanthin (peak 10) was found in the extracts in considerable amounts mostly in the ethanolic extract (5.50 mg/g DW). The early reports on *Nannochloropsis* carotenoids described that 40–58% of total carotenoids are violaxanthin derivatives, including its 5,8-furanoid isomers, 25–32% vaucheriaxanthin esters, 3–10% β-carotene, 0–1% neoxanthin, 4–5% keto-carotenoids, and 3–4% other minor carotenoids [7,56]. In accordance with those reports, in the present study, the derivatives of violaxanthin and its furanoid isomers (luteoxanthin and auroxanthin) are 61.6% in the ethanol extract and 76% in the BP extract. We did not detect vaucheriaxanthin esters and an explanation is that they were not extracted with those solvents, probably due to their lipophilicity. In other studies, saponification is employed in order to obtain the main carotenoids in high values [7]. In our study, antheraxanthin and its derivatives constitute a significant proportion of total carotenoids (29% in the ethanol extract and 15% in the BP) in accordance with the earlier studies, and the rest is β-carotene (6.2% in the ethanol and 3.2% in the BP extract). Canthaxanthin, the only keto-carotenoid, is also not quantified and we presume that traces of it co-elute in peak 7. Peak 7 is probably a mixture of two compounds, an auroxanthin derivative and canthaxanthin. Canthaxanthin is proposed due to the presence of a shift at 472 nm at the UV-vis spectrum and the ion with *m*/*z* 565.3, which corresponds to the molecular ion of canthaxanthin. This mixture of ingredients was quantifiable only at the ethanolic extract. The allenic epoxide, neoxanthin, that is produced from violaxanthin has also been reported in *Nannochloropsis* but, in this study, we could only observe a minor not quantified derivative (peak 3).

Luteoxanthin and auroxanthin are conversion products of violaxanthin; the two 5,6-epoxide groups in violaxanthin are converted to derivatives with one or two 5,8-furanoxide groups (luteoxanthin and auroxanthin, respectively). This rearrangement occurs in an acidic environment or after thermal treatment and is accompanied by a hypsochromic shift of about 20 nm per transition of the epoxide group [57,59,60,61]. The scenario that a portion of this structural rearrangement might have occurred during the prolonged cultivation and in the presence of acids that are produced in the cultivation or are present during the analysis cannot be overruled, but, most probably, those existed naturally in the microalgae. In unison, Owens et al. [7] supported the natural occurrence of the furanoid derivatives and showed age-dependent changes in the carotenoid composition; although the proportions of β-carotene and the vaucheriaxanthin group changed relatively little with the aging of *N. oculata*, the zeaxanthin-violaxanthin group tended to decrease, and both canthaxanthin and the astaxanthin family increased. The natural occurrence of luteoxanthin in Eustigmatophycae has also been reported in other studies summarized by Stoyneva-Gartner et al. [62].

Peak 14 with an absorption maximum at 432 nm corresponds to chlorophyll-a. The DAD detector in our experimental set-up scans from 190 to 640 nm, so the characteristic UV-vis maximum of chlorophylls at ~665 nm could not be recorded. Peak 15 was identified as pheophytin-a due to the UV-vis maximum at 408 nm and the polarity of the compound. It is expected for pheophytin-a, the demetalated chlorophyll-a, to elute later from a nonpolar C18 column as it is a less polar compound [63]. Additionally, the presence of the fragment ion with *m*/*z* 593.3, which corresponds to the loss of the phytyl chain as phytadiene (C_20_H_38_), confirms that claim [63]. Lastly, β-carotene (peak 16) was also present at 1.52 and 0.18 mg/g DW in ethanolic and BP extract, respectively, in other words, 6.2% and 3.2%. Lubian et al. [6] recorded a maximum value of 6% chlorophyll-a dry weight in *Nannochloropsis* sp.

**Figure 3:**
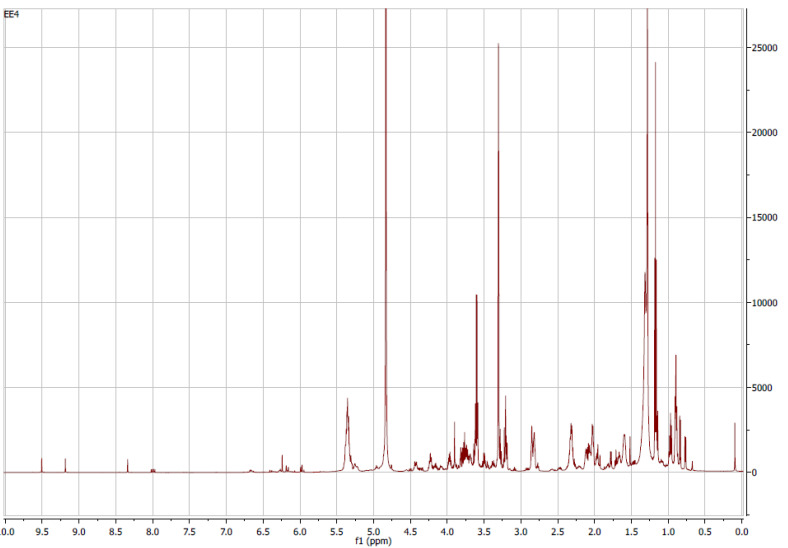
^1^H NMR spectrum (600.13 MHz) of *N. oculata* ethanolic extract metabolites in CD_3_OD.

The ^1^H-NMR fingerprinting of the EtOH extract was recorded to complement the characterization, since it is holistic and untargeted [64]. The signals from carotenoids and chlorophyll-a/pheophytin-a in the ethanolic extract could be observed (Figure 3). Violaxanthin-type carotenoids have a chain of trans double-linked conjugated bonds that correspond to signals in the range 6.7–6.0 ppm (Figure 3) [65]. According to the in-depth NMR study of Sobolev et al. [66], the spin system at 6.14, 6.34, and 6.64 is due to the fragment −(CH_3_)C=CH-CH=CH-C(CH_3_)− that is common to all carotenoids; corresponding shifts are observed in this study as well. The presence of chlorophyll-a and pheophytin-a is manifested by the singlet signals at 9.51, 9.18, and 8.34 ppm (Figure 3); those are attributed to the H-10, H-5, and H-20 methinic protons that bridge the pyrrole rings in porphyrin (Figure S3) [66]. In addition, the dd signal at 8.02–7.97 ppm is characteristic of H-3^1^, while the double at 6.41 ppm is attributed to H-3^2^ olefinic protons that are substituents to the pyrrole group (Figure 3 and Figure S3) [66]. The remaining signals of those compounds appear in the regions 3.7–0.8 (−CH_3_ and −CH_2_) and 4.5–3.9 (−CH, −COOCH_3_, and −COOCH_2_) (Figure 3). The peaks in the high field are congested and of high intensity. They belong not only to carotenoids, but also to amino acids, carbohydrates, and fatty acids [64]. Signals at the regions 4.0–3.0 ppm and 2.5–0.5 ppm (Figure 3) could correspond to amine protons, methyl, and methine groups of amino acids, whereas protons of carbohydrates appear at 3.0–4.2 ppm and the anomeric ones at higher ppm [64]. Glycerol moieties from triacylglycerides could also be present at 5.3, 4.3, and 4.1 ppm, while 4.0–3.0 ppm is the region where phospholipids can be detected [67,68]. Fatty acids can also be confirmed by the presence of shifts in the region 5.4–5.3 (olefinic protons) [64,68]. Figure 3 displays a multiple signal at 5.4 ppm, which could correspond to the protons of the double bonds of the unsaturated fatty acids. Another characteristic region for fatty acids is 2.8–0.9 ppm due to the protons of the allylic chains, those near the carboxyl ends, and aliphatic protons [68]. Thus, the fingerprinting analysis by ^1^H-NMR shows the presence of not only carotenoids and chlorophylls, but also amino acids, sugars, and fatty acids.

### 3.3. Characterization of the Microalgal Biomass before and after the Extraction

The original (not treated, NT) and the residual biomass were evaluated regarding their content in moisture, ash, proteins, carbohydrates, chlorophyll-a, carotenoids, and lipids, as well as fatty acid profile. The results are presented in Table 4 and Table 5.

Moisture content was low in the nontreated biomass (2.73 ± 1.73%) and practically zero in the biomass extracted with EtOH, without, however, a significant difference between the two treatments. Biomass treated with BP had a significantly higher moisture content (12.74 ± 1.86%) than both other conditions. That was possibly due to residual 1,2-propanediol registering as moisture after evaporating completely in the oven. The nontreated biomass formed aggregates that were moderately difficult to pulverize, an issue that was far more intense for the BP-treated biomass, with the formation of hard particles that were very difficult to break and not possible to pulverize by hand. On the other hand, the biomass treated with EtOH turned into a fine powder with minimal effort. That, in combination with the absence of moisture, makes EtOH a very appealing solvent, since industrial operations prefer dry and easy-to-handle bulk materials. The residual biomass would have a lower cost of drying due to the low evaporation temperature of H_2_O/EtOH azeotropes. It must be noted though that the extrusion process during the production of aquaculture feed requires a level of moisture between 10–20% depending on the desired density of the product [69].

The ash content differed significantly between the treatments, with EtOH-treated biomass having the highest content (8.91 ± 0.49). The carbohydrate content of the EtOH treatment (13.24 ± 0.54) was significantly higher than that of the BP and NT treatments. The EtOH-treated biomass also had the highest protein content (53.98 ± 2.93), which was not, however, significantly different from that of BP-treated biomass (49.16 ± 1.29). Lipids were still present in EtOH-treated biomass, although the content (3.86 ± 0.18) was much lower than that of NT- and BP-treated biomass, with the difference between the latter being also significant. Increased carbohydrate and protein after extraction of lipids from microalgae have been reported earlier [70], in agreement with the results presented here (Table 4).

The EtOH-treated biomass had significantly lower chlorophyll-a content than the other two treatments (1.30 ± 0.03), while the carotenoid and xanthophyll content of EtOH and BP treatments was significantly lower than that of nontreated biomass (0.21 ± 0.01 and 0.49 ± 0.02, respectively), but they were still present in the residual biomass along with the other valuable nutrients.

The fatty acid profile of the nontreated biomass was typical for *Nannochloropsis* [71,72], with palmitic acid (C16:0) and palmitoleic acid (C16:1) being the most abundant ones (22.78 ± 0.39% and 23.17 ± 0.52%, respectively) followed by EPA (C20:5n3) (20.68 ± 0.13%) (Table 5). Nontreated biomass had significantly higher pentadecenoic acid (C15:1) and linoleic acid (C18:2) fractions (1.43 ± 0.02% and 11.62 ± 0.2%) than the extracted biomass, while the EtOH-treated biomass had significantly higher 20:3n3 and significantly lower 20:5n5 FA fractions (5.57 ± 0.22% and 16.14 ± 0.33%, respectively) than the other two treatments (Table 5). A significant decrease in the total polyunsaturated fatty acid fraction was observed between treatments, with 38.16 ± 0.34% in the nontreated biomass, followed by 32.19 ± 1.01% in the BP-treated biomass and 26.42 ± 0.88 in the EtOH-treated biomass. The decrease in EPA in the EtOH treatment is also significant in terms of content on the DW basis, with 6.17 ± 2.78 mg EPA g^−1^ DW compared to 31.89 ± 0.86 mg EPA g^−1^ DW in nontreated biomass and 27.03 ± 2.55 mg EPA g^−1^ DW in BP-treated biomass. The total unsaturated FA content also had significant differences between the treatments, with EtOH having the lowest content (Table 5).

## 4. Conclusions

Different extraction methods were evaluated by assessing their reactivity with Folin-Ciocalteu and UAE extraction, with ethanol and the natural deep eutectic solvent BP being selected as those giving high yields without depleting the biomass; this is the first report on the neoteric NaDES for *Nannochloropsis* extraction. Our findings on the pigment extraction efficiency of enzymic treatments, aqueous solutions of different pH values, and cyclodextrins will also be useful to other researchers since most previous investigations were focused on lipids and some of those treatments have not been evaluated in microalgae. An HPLC-DAD method was developed for the separation, characterization, and quantification of the carotenoids, with the results being in agreement with the literature. The ethanol extract had a higher antioxidant capacity and a higher carotenoid content, although the extract also contained chlorophyll-a, pheophytin, amino acids, carbohydrates, and fatty acids, as evidenced by ^1^H-NMR. That was corroborated by the study of the biomass before and after the extraction. The ethanol treatment caused the largest depletion of fatty acids, although they were still present in the remaining biomass, which contained the highest content of proteins and carbohydrates and was completely dry and easy to handle. Therefore, UAE extraction with ethanol firstly and BP secondly seem to serve the dual aim of our investigation to obtain “green” antioxidant extracts and a nutritious residue of *Nannochlorpsis oculata* for aquafeed. Although further specific experiments on the value of the obtained extracts and biomasses in cosmetology and aquaculture are necessary, and optimization of the processes for large-scale production can be performed, our study greatly contributes to the quest for sustainable valorization of *Nannochloropsis oculata*.

## Figures and Tables

**Table 1:** List of different extraction methods of *N. oculata*. Details on the solvents, solvent-to-biomass ratio, temperature, and time of extraction are provided. Superscript numbers provide the earlier references of the extraction methods, which were used as they were or after minor modification.

Extract Abbreviation	Procedure	Solvent	T (°C)	Duration
W UAE	UAE	water (2 mL/40 mg, four times with the same biomass)	<40	15 min each extraction step, four repetitions (total 60 min)
MeOH UAE	UAE	methanol (2 mL/40 mg, four times with the same biomass)	<40	15 min each extraction step, four repetitions
EtOH UAE	UAE	ethanol (2 mL/40 mg, four times with the same biomass)	<40	15 min each extraction step, four repetitions
70%EtOH UAE	UAE	70% ethanol (2 mL/40 mg, four times with the same biomass)	<40	15 min each extraction step, four repetitions
AcOH/70%EtOH UAE	UAE	1% acetic acid in 70% ethanol (2 mL/40 mg, four times with the same biomass)	<40	15 min each extraction step, four repetitions
EtOH RT	Maceration	ethanol (2 mL/40 mg, four times with the same biomass)	room temperature	15 min each extraction step, four repetitions
EtOH 50 °C	Maceration under heating	ethanol (2 mL/40 mg, four times with the same biomass)	50	15 min each extraction step, four repetitions
EtOH 80 °C	Maceration under heating	ethanol (2 mL/40 mg, four times with the same biomass)	80	15 min each extraction step, four repetitions
ALCALASE	Enzymatic treatment [38]	0.4% of Alcalase^®^ diluted in 0.1 M phosphate buffer, pH 7.0 (1.25 mL/40 mg)	47	2 h
VISCOZYME	Enzymatic treatment [38]	0.4% of Viscozyme^®^ diluted in 0.1 M acetate buffer, pH 4.5 (1.25 mL/40 mg)	47	2 h
pH 4.5 (serves as control for VISCOZYME)	Maceration under heating	0.1 M acetate buffer, pH 4.5 (1.25 mL/40 mg)	47	2 h
pH 7.0 (serves as control for ALCALASE)	Maceration under heating	0,1 M phosphate buffer, pH 7.0 (1.25 mL/40 mg)	47	2 h
pH 9.0	Maceration under heating	alkalized water at pH 9.0 (1.25 mL/40 mg)	47	2 h
βCD W	UAE [32,33]	1.5% of aqueous β-cyclodextrin (2 mL/ 40 mg) ^a^	<40	1 h
W control (serves as control for βCD W)	UAE [32,33]	1.5% of water (2 mL/ 40 mg) ^a^	<40	1 h
βCD 50%EtOH	UAE [32,33]	1.5% of β-cyclodextrin in 50% EtOH (2 mL/ 40 mg) ^a^	<40	1 h
50% EtOH control (serves as control for βCD 50% EtOH)	UAE [32,33]	50% EtOH (2 mL/ 40 mg) ^a^	<40	1 h
BP	UAE [39]	betaine: 1,2-propanediol (2:5, molar ratio) with the addition of water to the final 12.5% (0.4 mL/40 mg biomass and dilution in 1 mL water)	<40	40 min
BG	UAE [40]	betaine: glycerol (1:2, molar ratio) with the addition of water to the final 12.5% (0.4 mL/40 mg biomass and dilution in 1 mL water)	<40	40 min
BGG4	UAE [41]	betaine: glycerol: glucose (4:20:1, molar ratio) with the addition of water to the final 12.5% (0.4 mL/40 mg biomass and dilution in 1 mL water)	<40	40 min
UG	UAE [42]	urea: glycerol (1:1, molar ratio) with the addition of water to the final 12.5% (0.4 mL/40 mg biomass and dilution in 1 mL water)	<40	40 min

^a^ Steps as follows: i. centrifugation, ii. freeze-drying of the residue, iii. dilution in 1 mL ethanol, iv. centrifugation, and v. collection of the supernatant. Steps iii–v were repeated twice. Final volume in ethanol 2 mL.

**Table 2:** Antioxidant capacity of the two extracts.

Extract	Folin–Ciocalteu mg GAE/g DW	FRAP mg TEQ/g DW	DPPH mg TEQ/g DW	ABTS mg TEQ/g DW
EtOH-UAE	5.39 ± 0.23 ^a^	23.46 ± 1.96 ^a^	5.42 ± 0.20 ^a^	4.15 ± 0.03 ^a^
BP	3.68 ± 0.12 ^b^	6.73 ± 0.09 ^b^	2.27 ± 0.21 ^b^	2.04 ± 0.01 ^b^

Different letters (a, b) as superscripts in the same column show difference in statistical significance at *p* < 0.05.

**Table 3:** Identification and quantification of the main compounds of the two extracts, the EtOH-UAE and the BP. Peaks were tentatively identified after comparison of the UV-vis maxima obtained by the HPLC-DAD and UHPLC-DAD-MS analysis, the ions observed in the mass spectra after UHPLC-DAD-MS, and their elution order to the literature. The retention times of the HPLC-DAD analysis used for quantification are presented. The concentrations (mean ± standard deviation, *n* = 3) are presented as mg astaxanthin equivalents per g dry original microalgal biomass.

Peak Number	Tentative Identification	Rt (min)	MW	UV-Vis Maxima (nm)	Positive Ionization (*m*/*z*)	EtOH-UAE	BP
1	violaxanthin derivative [8]	6.67	618.9	417, 441, 470	619.5 [M+H]^+^ 601.5 [M−18+H]^+^ 583.5 [M−18−18+H]^+^	0.94 ± 0.06	0.53 ± 0.04
2	luteoxanthin derivative	7.84	618.9	400, 423, 450	619.5 [M+H]^+^ 601.5 [M−18+H]^+^ 583.5 [M−18-−18+H]^+^	nq	nq
3	neoxanthin derivative [54]	8.64	618.9	412, 435, 465	619.5 [M+H]^+^ 601.4 [M−18+H]^+^ 583.5 [M−18−18+H]^+^	nq	nq
4	violaxanthin [8,54,55]	9.12	600.9	417, 441, 470	601.5 [M+H]^+^ 584.5 [Μ−18+H]^+^ 583.4 [Μ−18]^+^	10.41 ± 0.94	2.34 ± 0.23
5	luteoxanthin derivative [7]	10.32	616.9	400, 423, 450	601.6 [Μ−15]^+^ 583.6 [M−18−15]^+^ 617.5 [Μ+H]^+^	1.35 ± 0.07	0.55 ± 0.04
6	luteoxanthin derivative [7]	10.54	616.9	400, 423, 450	601.6 [Μ−15]^+^ 583.6 [M−18−15]^+^ 617.5 [Μ+H]^+^	1.39 ± 0.08	0.60 ± 0.03
7	auroxanthin derivative + canthaxanthin [7,56]	11.64	614.9 (M_1_) & 564.9 (M_2_)	381, 402, 427 (sh472)	615.4 [M_1_+H]^+^ 599.6 [M_1_−15]^+^ 584.6 [M_1_−15−15]^+^ 565.3 [M_2_+H]^+^	0.52 ± 0.02	nq
8	cis-hydroxylated carotenoid [54]	12.36	600.9	315, 330, 417, 440, 464	601.5 [M+H]^+^ 584.5 [M−18+H]^+^	0.84 ± 0.06	0.30 ± 0.02
9	auroxanthin [7,57]	12.92	600.9	381, 402, 427	601.6 [M+H]^+^ 583.6 [M−18]+	0.49 ± 0.03	0.18 ± 0.01
10	antheraxanthin [7,8,54,57]	15.12	584.9	420, 444, 472	585.5 [M+H]^+^	5.50 ± 0.49	0.59 ± 0.06
11	Luteoxanthin [7,57]	18.20	600.9	400, 424, 452	601.5 [M+H]^+^ 584.5 [M−18+H]^+^]^+^	nq	nq
12	antheraxanthin derivative [8] (propionyl ester?)	19.15	640.9	420, 445, 472	641.5 [M+H]^+^ 623.6 [M−18+H]^+^	1.54 ± 0.11	0.26 ± 0.02
13	cis-carotenoid [54] (9-cis-violaxanthin isomer)	19.85		330, 418, 438, 466		nq	nq
14	chlorophyll-a [7,8]	28.85	893.5	432		-	-
15	pheophytin a [58]	41.21	871.2	408	593.3 [M−C_20_H_38_]^+^	-	-
16	β-carotene [7,8,54,55]	41.85	536.9	450, 480	537.5	1.52 ± 0.14	0.18 ± 0.01

The previous references that helped in the identification are provided in the second column, and, in the parentheses, a suggestion for the nature of the identified compound is given. nq: not quantified.

**Table 4:** Humidity after freeze-drying and proximate composition in dry weight basis of nontreated and residual biomass after EtOH-UAE and BP extraction.

	NT	ETOH-UAE	BP
Humidity%	2.73 ± 1.73 ^a^	−0.40 ± 1.23 ^a^	12.16 ± 0.96 ^b^
Ash%	5.88 ± 0.18 ^a^	8.91 ± 0.49 ^b^	7.81 ± 0.17 ^c^
Carbohydrates%	9.20 ± 1.57 ^b^	13.24 ± 0.54 ^a^	9.05 ± 1.56 ^b^
Proteins%	43.66 ± 7.13 ^a^	53.98 ± 2.93 ^b^	49.16 ± 1.29 ^b^
Chlorophyll-a%	4.96 ± 0.24 ^a^	1.30 ± 0.03 ^b^	2.27 ± 1.31 ^a^
Carotenoids%	1.27 ± 0.06 ^a^	0.21 ± 0.01 ^b^	0.49 ± 0.02 ^b^
Lipids%	16.00 ± 0.44 ^a^	3.86 ± 0.180 ^b^	12.74 ± 1.86 ^c^
Others	19.03 ± 9.62	18.50 ± 7.04	18.48 ± 5.99

Different letters (a, b, c) as superscripts within the same row represent significant differences at the *p* < 0.05 level.

**Table 5:** Fatty acid profile and content on dry weight (DW) basis for nontreated and residual biomass after EtOH and BP extraction.

Fatty Acids	No treatment		EtOH		BP	
	% DW	% FAs	% DW	% FAs	% DW	% FAs
C10:0	-	-	-	-	-	-
C11:0	-	-	-	-	-	-
C12:0	-	-	-	-	-	-
C13:0	-	-	-	-	-	-
C14:0	11.43 ± 0.12 ^a^	7.41 ± 0.07	3.27 ± 1.30 ^b^	8.81 ± 0.74	9.86 ± 0.41 ^a^	7.77 ± 0.41
C14:1	1.73 ± 1.73	1.1 ± 1.1	0.74 ± 0.74	1.31 ± 1.31	-	-
C15:0	-	-	-	-	-	-
C15:1	2.21 ± 0.02	1.43 ± 0.02	-	-^b^	-	-^b^
C16:0	35.11 ± 0.13 ^a^	22.78 ± 0.39	9.85 ± 4.23 ^b^	26.13 ± 1.26	30.16 ± 2.17 ^a^	23.72 ± 0.55
C16:1	36.72 ± 0.06 ^a^	23.17 ± 0.52	9.26 ± 4.30 ^b^	24.04 ± 0.09	31.86 ± 2.31 ^a^	25.06 ± 0.57
C17:0	-	-	-	-	-	-
C17:1	-	-	-	-	-	-
C18:0	-	-	-	-	-	4.15 ± 4.15
C18:1	9.17 ± 0.17	5.95 ± 0.02	4.07 ± 1.55	11.1 ± 1.18	8.66 ± 3.25	7.1 ± 3.23
C18:2	17.91 ± 0.06 ^a^	11.62 ± 0.20	1.88 ± 0.98 ^b^	4.71 ± 0.33	7.29 ± 0.74 ^b^	5.71 ± 0.04
C18:3n6	-	-	-	-	-	-
C18:3n3	-	-	-	-	-	-
C20:0	-	-	-	-	-	-
C20:1	-	-	-	-	-	-
C20:2	2.57 ± 0.04	1.66 ± 0.01	-	-	1.07 ± 1.07	0.77 ± 0.77
C20:3n6	-	-	-	-	-	-
C21:0	-	-	-	-	-	-
C20:3n3	6.49 ± 0.13 ^a^	4.21 ± 0.01	2.11 ± 0.92 ^b^	5.57 ± 0.22	5.71 ± 0.31 ^a^	4.5 ± 0.18
C20:4n6	-	-	-	-	--	-
C20:5n3	31.89 ± 0.86 ^a^	20.68 ± 0.13	6.17 ± 2.78 ^b^	16.14 ± 0.33	27.03 ± 2.55 ^a^	21.21 ± 0.02
C22:0	-	-	-	2.19 ± 2.19	-	-
C22:1n9	-	-	-	-	-	-
C22:2	-	-	-	-	-	-
C23:0	-	-	-	-	-	-
C24:0	-	-	-	-	-	-
C22:6n3	-	-	-	-	-	-
C24:1	-	-	-	-	-	-
Total	154.22 ± 3.32 ^a^	100	38.59 ± 18.04 ^b^	100	127.43 ± 18.6 ^c^	100
MUFAs	48.82 ± 1.97 ^a^	31.65 ± 1.65	14.07 ± 6.59 ^b^	36.45 ± 2.58	40.53 ± 5.56 ^c^	32.16 ± 3.79
PUFAs	58.85 ± 1.09 ^a^	38.16 ± 0.34	10.15 ± 4.68 ^b^	26.42 ± 0.88	41.1 ± 4.67 ^c^	32.19 ± 1.01

Different letters (a, b, c) as superscripts within the same row represent significant differences at the *p* < 0.05 level. MUFAs: monounsaturated fatty acids; PUFAs: polyunsaturated fatty acids.

## Data Availability

The data presented in this study are available in the article and supplementary materials.

## Supplementary Materials

The following supporting information can be downloaded at: https://www.mdpi.com/article/10.3390/antiox11061103/s1, Figure S1: UV-vis spectrum of ALCALASE Extract. Figure S2: Typical HPLC chromatogram at 280 nm of the EtOH-UAE extract. Figure S3: Structure of pheophytin a.
