# Supplementary material for: Ultrasound-Assisted Extraction of Nannochloropsis oculata with Ethanol and Betaine: 1,2-Propanediol Eutectic Solvent for Antioxidant Pigment-Rich Extracts Retaining Nutritious the Residual Biomass
Source: Antioxidants (Basel). 2022 May 31;11(6):1103. doi: 10.3390/antiox11061103 (PMC9220189; doi:10.3390/antiox11061103)
Supplement: Supplementary file 1 [file antioxidants-11-01103-s001.zip › antioxidants-1725526-supplementary.pdf]

SUPPLEMENTARY FIGURES

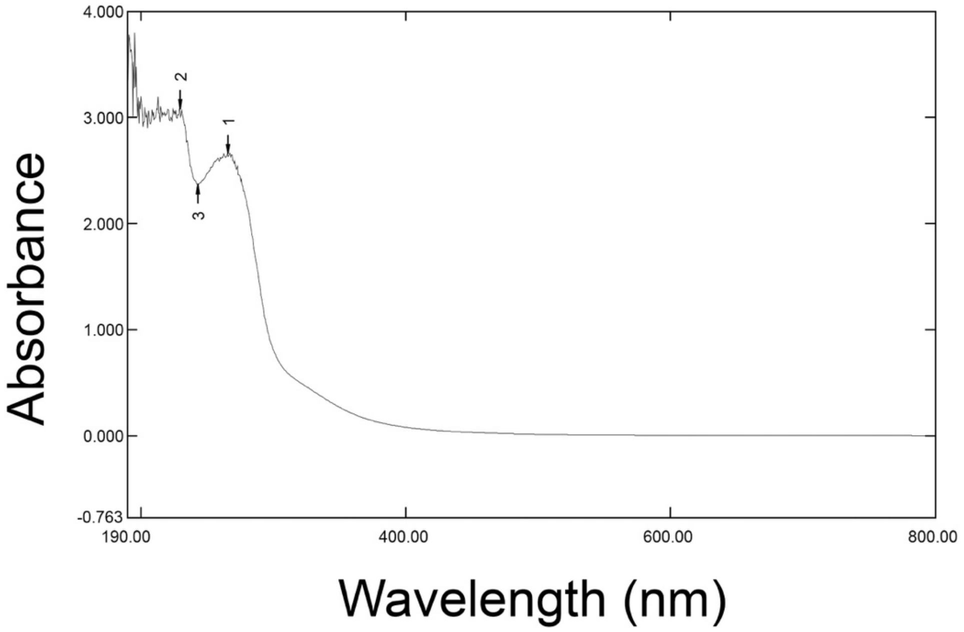

Figure S1. UV-vis spectrum of ALCALASE Extract.

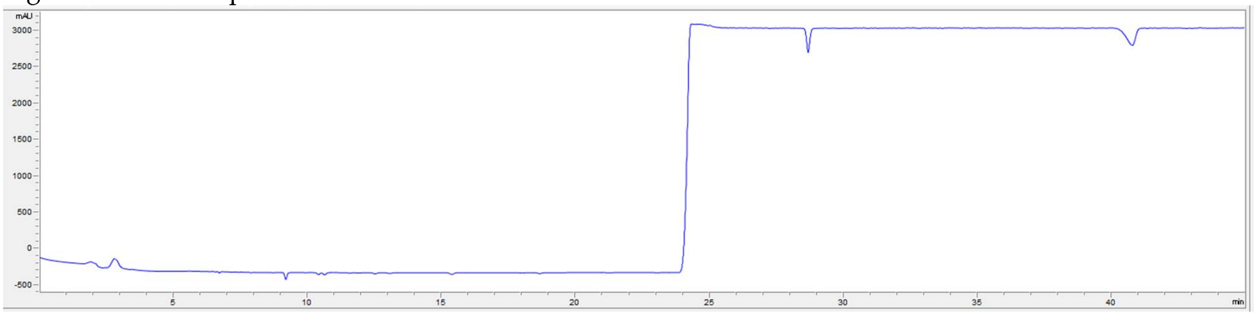

Figure S2. Typical HPLC chromatogram at 280 nm of the EtOH-UAE extract.

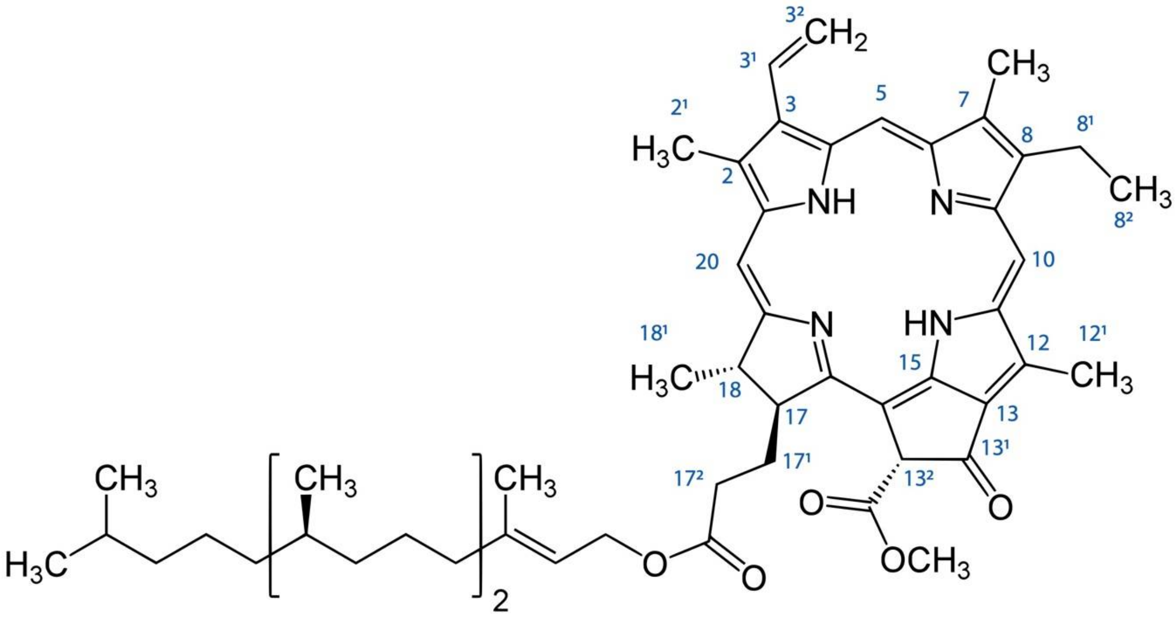

Figure S3. Structure of pheophytin a.
